# Flower‐Doped Carbon Quantum Dots Improve *Ceratobasidium* sp. Growth Efficiency: A Green Nanotechnology Strategy for Fungal Applications

**DOI:** 10.1111/1758-2229.70382

**Published:** 2026-06-30

**Authors:** Bengisu Sentürk, Erdi Can Aytar, Major Mabuza, Idara Asuquo Okon, Mika Sillanpää, Yasemin Özdener Kömpe

**Affiliations:** ^1^ Faculty of Science, Department of Biology Ondokuz Mayıs University Samsun Turkey; ^2^ Faculty of Agriculture, Department of Horticulture University of Usak Usak Turkey; ^3^ Department of Chemical Engineering Technology, Faculty of Engineering and the Built Environment University of Johannesburg Johannesburg Republic of South Africa; ^4^ Department of Physiology, Faculty of Biomedical Sciences Kampala International University Kampala Bushenyi Uganda; ^5^ Key Laboratory of Northwest Water Resource, Environment and Ecology, MOE Xi'an University of Architecture and Technology Xi'an China; ^6^ Institute for Nanotechnology and Water Sustainability (iNanoWS), Florida Campus, College of Science, Engineering and Technology, University of South Africa Johannesburg South Africa; ^7^ Centre of Research Impact and Outcome Chitkara University Institute of Engineering and Technology, Chitkara University Rajpura Punjab India

**Keywords:** *Ceratobasidium*, fungal biotechnology, growth rate optimization, nanomaterials in mycology, quantum dots

## Abstract

This study investigates the characteristics of AL (*Anacamptis laxiflora* flower)‐doped carbon quantum dots (AL‐CQDs), synthesised via a 7‐h pyrolysis process and their effects on the growth of the *Ceratobasidium* sp. (AL5) fungus. AL‐CQDs exhibited strong ultraviolet–visible absorption and a consistent spherical morphology. High‐resolution transmission electron microscopy (HR‐TEM) analysis revealed that the particles were homogeneously distributed, predominantly spherical or quasi‐spherical in shape and ranged in size from 45 to 110 nm. In addition, the HR‐TEM images showed localised ordered domains with an average interplanar spacing of approximately 0.113 nm, indicating the presence of graphitic or crystalline‐like regions within the carbon dots. Fungal growth experiments demonstrated that nutrient media supplemented with CQDs significantly increased the growth rate and decreased hyphal diameter, suggesting more efficient nutrient uptake. The medium containing 2 g of CQDs promoted the fastest growth, reaching maximum levels by Day 5, whereas the 1 g CQD medium resulted in a more compact cell structure and steady growth. These findings suggest that AL‐CQDs enhance fungal growth and may have promising applications in fungal biotechnology and nanobiotechnology.

## Graphical abstract

Carbon quantum dots (AL‐CQDs) synthesised from *Anacamptis laxiflora* were characterised by HR‐TEM, FT‐IR and UV–Vis spectroscopy and evaluated for their effects on the mycorrhizal fungus *Ceratobasidium* sp. (AL5). AL‐CQDs significantly influenced fungal growth and hyphal morphology, highlighting their potential in plant–fungus interaction studies.

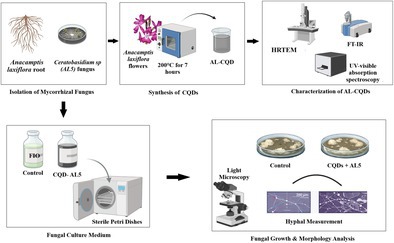

## 1 Introduction

Carbon quantum dots (CQDs) are zero‐dimensional nanostructures with a carbon core, having all dimensions less than 10 nm. These nanostructures, which have a hemispherical shape, were serendipitously discovered in 2004 during the purification of single‐walled carbon nanotubes (SWCNTs). Despite the inherently low solubility of carbon in water, CQDs exhibit remarkable water solubility due to the abundant carboxyl groups at their edges (Pourmadadi et al. 2023). In comparison to other zero‐dimensional carbon‐based nanomaterials such as graphene quantum dots (GQDs), CQDs possess a lower content of crystalline sp^2^ carbon and exhibit a higher density of surface defects. As a result of these properties, CQDs demonstrate less pronounced crystallinity relative to GQDs. The distinctive structural characteristics of CQDs enable them to function as both electron donors and acceptors, thereby facilitating their application in photocatalytic systems (Dananjaya et al. 2024).

In recent years, carbon quantum dots (CQDs) have attracted considerable attention owing to their strong photoluminescence, tunable fluorescence emission, high photochemical stability, excellent water solubility and remarkable biocompatibility. Their low toxicity and eco‐friendly nature further support their suitability for biomedical and clinical applications. Moreover, the presence of abundant surface functional groups such as carboxyl, carbonyl, hydroxyl and amino groups enables facile functionalization, thereby enhancing sensitivity, selectivity and optical tunability. In addition to their favourable physicochemical stability and conductivity, CQDs are distinguished by their low production cost and simple synthesis processes, which make them highly attractive for diverse technological and biomedical applications (Mahto et al. 2024; Zou et al. 2024; Wu et al. 2024).

Endophytes are microorganisms that reside in the intercellular spaces of healthy plant tissues without causing any disease. They provide nutrients to the host, enhance plant resistance to pathogens, cold and drought, and support plant growth by secreting hormones or supplying nutrients (Sallam et al. 2021). Many fungi belonging to Ascomycota and Basidiomycota, typically existing as saprotrophs in soil, also establish root‐associated interactions such as ectomycorrhizal and endophytic relationships. These interactions may range from mutualistic to commensal or pathogenic, depending on environmental conditions and host physiology (Hossain 2022).

Mycorrhiza represents a form of biotrophic symbiosis that is widespread in more than 90% of terrestrial plants, facilitating nutrient exchange and enhancing plant fitness (Andrade et al. 2024). In orchids, this relationship is particularly critical, as seed germination and early development strictly depend on fungal partners. Although *Ceratobasidium* species are frequently associated with orchid mycorrhizal systems, they are not exclusively obligate mycorrhizal fungi; rather, they comprise ecologically versatile taxa that may occur as orchid symbionts, endophytes or members of the *Rhizoctonia* complex depending on host identity and environmental context (Mosquera‐Espinosa et al. 2013; Otero et al. 2002). In contrast to arbuscular mycorrhizal fungi, which are generally regarded as obligate biotrophs, *Ceratobasidium* isolates can be maintained under axenic culture conditions, supporting their facultative, endophytic‐like lifestyle (Freestone et al. 2022; Tanaka et al. 2022; Wipf et al. 2019). In non‐orchid hosts, some *Ceratobasidium* taxa may behave as pathogens and cause root or stem diseases, whereas in orchid systems they often form non‐pathogenic associations that promote seed germination, nutrient transfer, stress tolerance and plant development (Durán‐López et al. 2019; Li et al. 2025; Pujasatria et al. 2024).

Orchid mycorrhiza, a specialised symbiotic system unique to Orchidaceae, plays a crucial role in plant survival, ecological adaptation and conservation. Symbiotically propagated orchid seedlings have shown higher success rates in ex vitro conditions, highlighting the importance of fungal partners in orchid biology and restoration strategies (Aytar and Kömpe 2024; Deniz et al. 2022).

The aim of this study is to investigate the effects of carbon quantum dots (CQDs) on the growth and development of AL5 (MT605389), an orchid‐associated fungal isolate identified as *Ceratobasidium* sp. Although *Ceratobasidium* species are commonly associated with orchid mycorrhizal systems, they are not obligate symbionts and are capable of growth under axenic culture conditions. Within orchid systems, these fungi generally form non‐pathogenic associations that may support host development, nutrient acquisition and adaptation to environmental stress. Although orchid–fungus interactions have been extensively recognised as biologically important, the potential of CQDs to modulate fungal growth and function has remained largely unexplored. Accordingly, this study was designed to evaluate the effects of CQDs on the growth characteristics of *Ceratobasidium* sp., with particular attention to their possible role in shaping fungus‐mediated plant–microbe interactions. The results may expand current understanding of nanomaterial applications in plant–fungus systems and support the development of novel agricultural and biotechnological approaches.

## 2 Material and Methods

### 2.1 The Isolation of Mycorrhizal Fungus

The isolation of *Ceratobasidium* sp. (AL5) fungus was conducted by Kömpe and Mutlu (2021). The isolation process involved collecting root samples of *Anacamptis laxiflora* under sterile laboratory conditions and transferring the mycorrhizal fungus from these roots into a culture medium. The isolated fungus was identified using molecular biology techniques and registered in the genetic database under the code MT605389. In this study, the germination rate of seeds symbiotically associated with the AL5 fungus was recorded at 59%.

### 2.2 Synthesis of Carbon Quantum Dots (CQDs)

In this study, the pyrolysis method was employed using *Anacamptis laxiflora* flowers. During the synthesis process, flower samples, each weighing 2 g, underwent pyrolysis at 200°C for 7 h. Following this procedure, 20 mL of pure water was added to each sample and the resulting carbon CQDs were extracted for 30 min. Subsequently, the samples were centrifuged at 5000 rpm for 15 min to separate the extract. The obtained extract was carefully filtered using a glass fibre filter with a pore size of 1.0 μm and a diameter of 25 mm to remove any remaining impurities. Finally, the filtered extract was stored at room temperature and preserved appropriately for future use. This sample was designated as AL‐CQD.

### 2.3 Characterisation of Carbon Quantum Dots (AL‐CQDs)

Fourier‐transform infrared spectroscopy (FT‐IR, Shimadzu) was utilised to determine the functional groups present in the obtained CQDs. UV–visible absorption spectroscopy (Perkin Elmer) was employed to ascertain the excitation wavelength of the CQDs. Additionally, high‐resolution transmission electron microscopy (HR‐TEM) analyses were performed on the samples using a Talos F200S scanning/transmission electron microscope (S/TEM) operating at a maximum acceleration voltage of 200 kV.

### 2.4 Addition of Quantum Dots to Fungal Culture Medium

The Fungus Isolation Medium (FIO) used for the isolation of mycorrhizal fungi from orchid roots was prepared according to the studies of Clements et al. (1986). The composition of this medium includes: 0.5 g of Calcium Nitrate (Ca(NO_3_)_2_·4H_2_O), 0.2 g of Potassium Dihydrogen Orthophosphate (KH_2_PO_4_), 0.1 g of Potassium Chloride (KCl), 0.1 g of Magnesium Sulphate (MgSO_4_), 0.5 g of Yeast Extract, 5 g of Sucrose and 10 g of Agar. The pH of the medium was adjusted to between 5.6 and 5.8. Apart from the normal FIO medium, variations were prepared by adding carbon quantum dots in amounts of 0.5, 1 and 2 g instead of sucrose as the carbon source. Additionally, formulations containing both sucrose and quantum dots (1 g each) were also prepared. The prepared solution intended for laboratory use was filled into glass bottles, and an equal volume of distilled water was added. The total volume of the solution components was adjusted to 1000 mL, and the pH was set to 5.8. Subsequently, Sucrose (Bioshop) and Agar (Sigma Aldrich) were added to the solution. The prepared nutrient medium was sterilised in an autoclave (NUVE LN 60) at 121°C for 20 min. After sterilisation, the nutrient medium was poured into sterile petri dishes (ISOLAB 90/17 mm) in a sterile laminar flow cabinet (NUVE LN 90).

### 2.5 Fungal Growth Rate and Morphology Analysis

The prepared nutrient media were inoculated with AL5 fungi. Growth rates were determined on Days 1 and 6, with additional measurements taken every 12 h on intervening days. Images and measurements of hyphal diameters formed in different environments were captured using a modified light microscope (DM4000 B; Leica), equipped with a motorised specimen stage for automatic sampling (Bio Precision MAC 5000 controller system; Ludl Electronic Products, Hawthorne, New York, USA) and a CCD colour video camera (Optronics Micro Fire, Goleta, California, USA).

### 2.6 Statistical Analysis

For evaluating the measurements of fungal hyphal diameters, an ANOVA test was employed due to the normal distribution and homogeneity of variances in the data. Duncan's test was used as a pairwise post hoc test to determine differences between species for each feature. Statistical analyses were conducted using the SPSS software package for Windows (version 21) (IBM Corp 2012).

## 3 Results

### 3.1 Characterisation of the Synthesised AL‐CQDs

**FIGURE 1:**
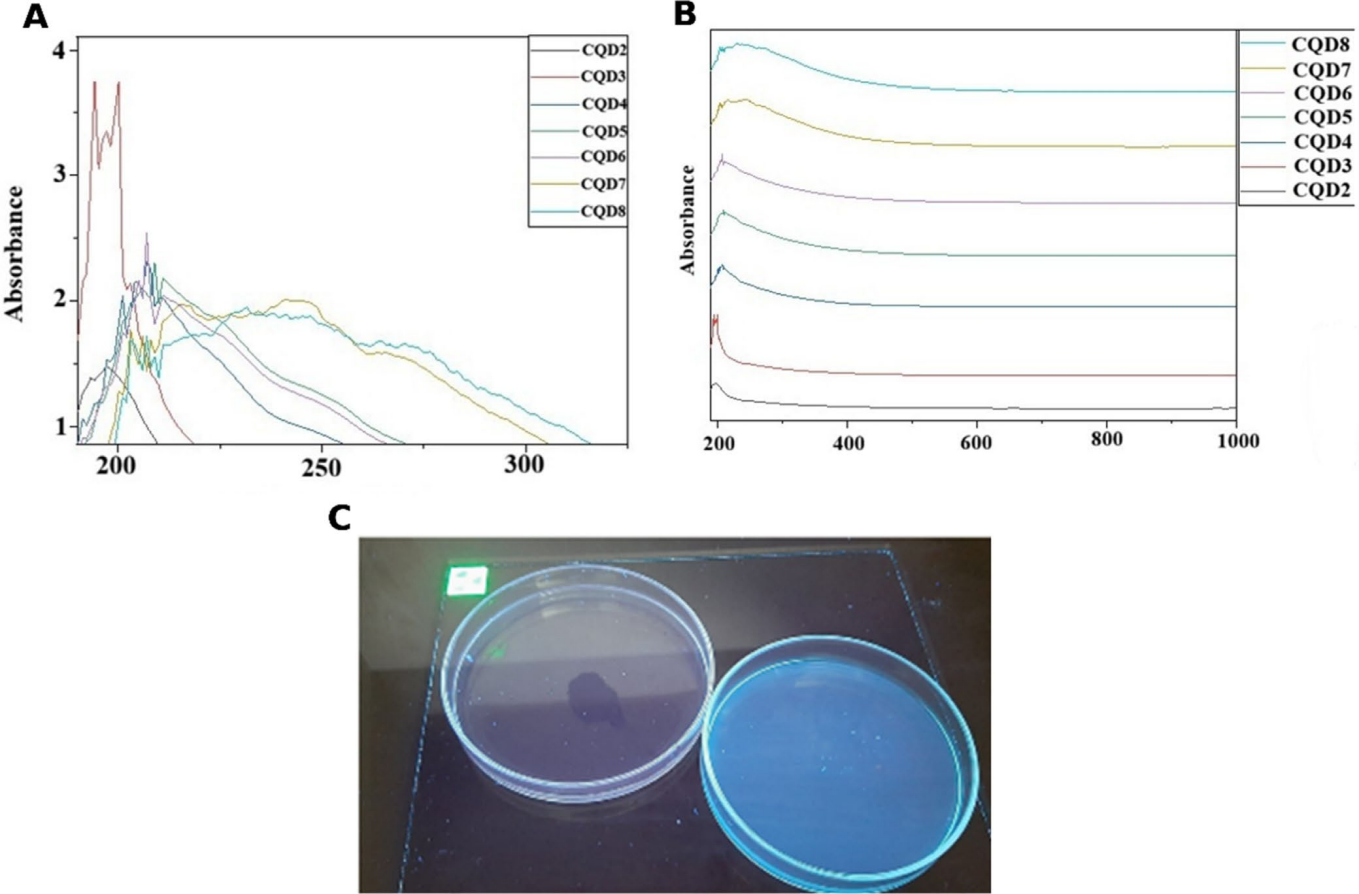
Optical characterisation of AL‐CQDs. (A) UV–Vis absorption spectra of AL‐CQDs synthesised at different pyrolysis times. (B) Absorption profiles of AL‐CQDs in the 200–1000 nm range. (C) Photograph of AL‐CQDs under UV illumination showing blue fluorescence.

**FIGURE 2:**
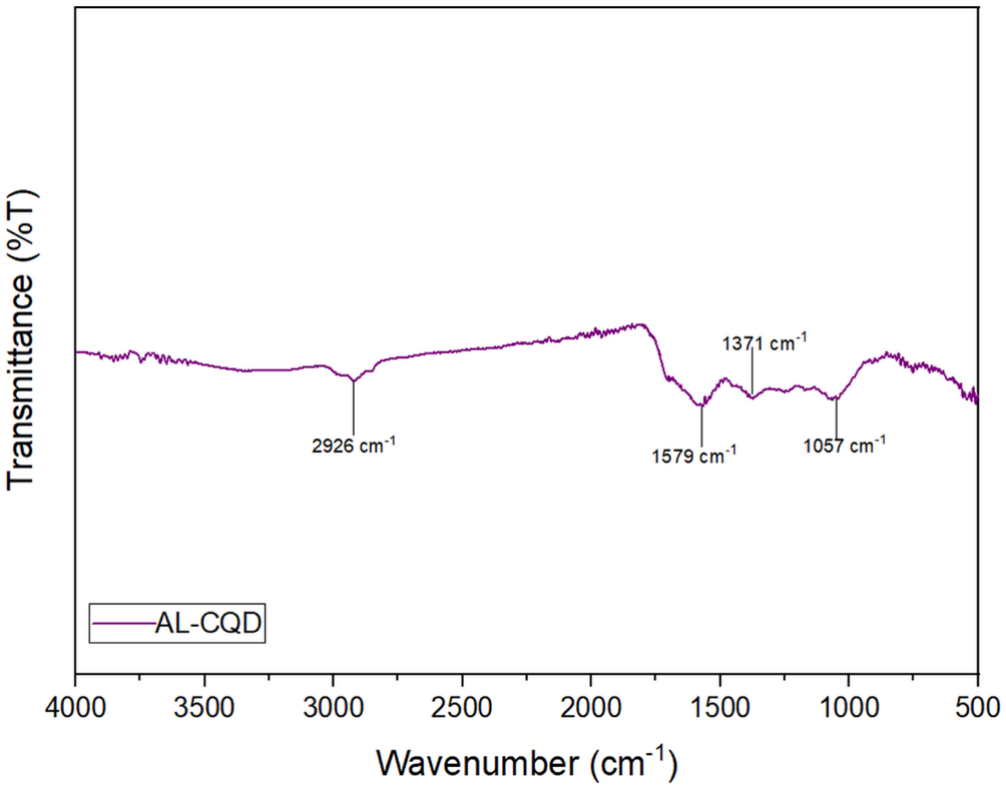
FTIR spectrum of the AL‐CQDs obtained from *A. laxiflora* flower.

The absorption spectrum results of synthesised AL‐CQDs samples are shown covering the visible–ultraviolet range. It is evident that a high absorption value is obtained after 7 h of pyrolysis (Figure 1). Based on this observation, all subsequent analysis and characterisation procedures were performed using samples of AL‐CQDs synthesised in a 7‐h pyrolysis process. With premium conditions in hand, for comparison, CQDs were obtained from plant extract. FTIR spectra obtained from various parts of the plant were shown in Figure 2 and Table 1. Almost the same spectra were observed for all the sources.

**TABLE 1:** General band assignments of the average FTIR spectrum.

Wavenumber cm^−1^	Functional group	Structure	References
2926	Methyl group	C–H	Aytar et al. (2023)
1579	Carboxyl groups	C–O	Mohamad Sukri et al. (2023)
1371	Carboxyl groups, aromatic ring C=C	–COOH C=C aromatic	Djebara et al. (2012)
1057	Stretch of C–O–H	C–O–H	Diwan et al. (2024)

**FIGURE 3:**
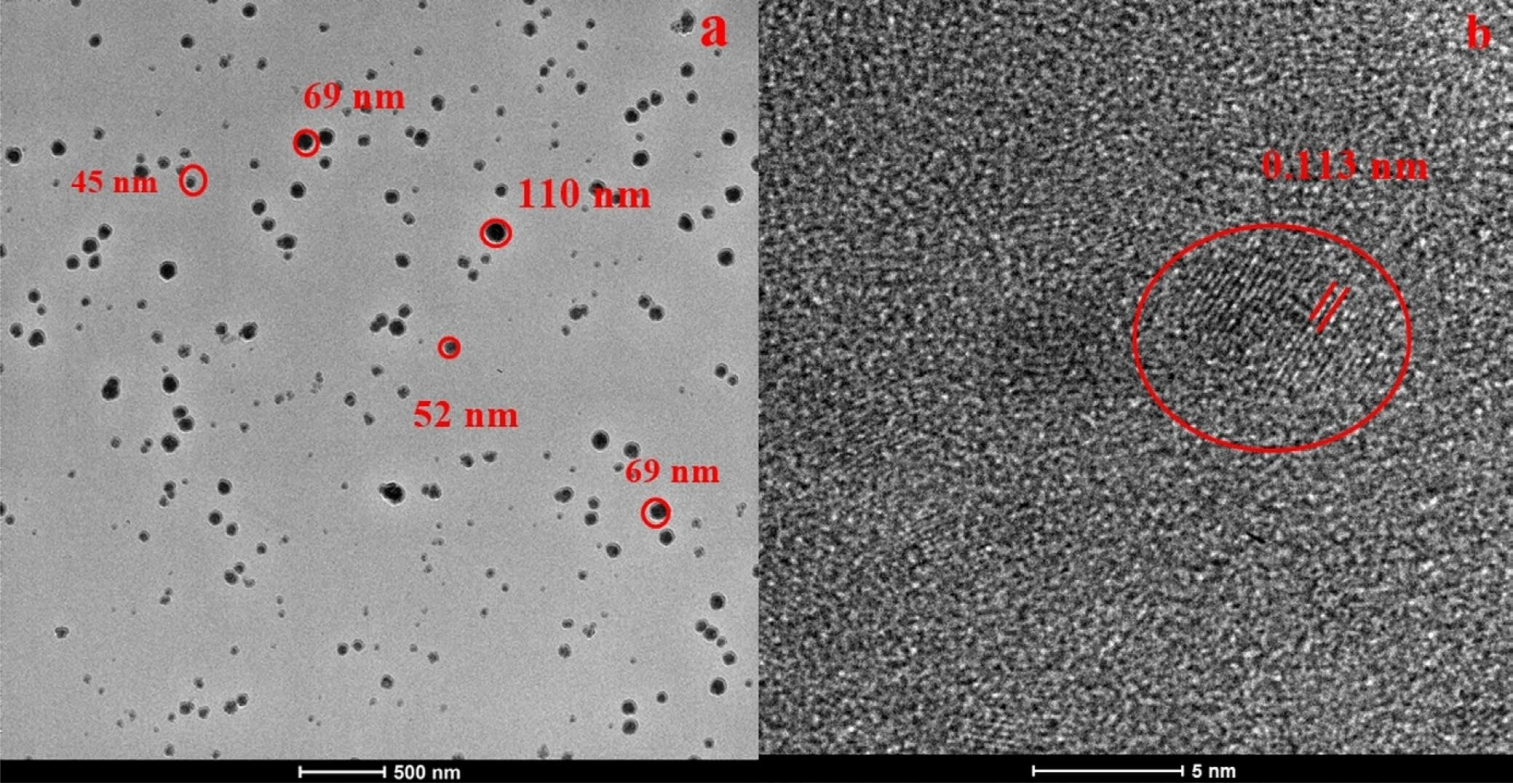
HR‐TEM images of the as‐prepared AL‐CQDs synthesised from *Anacamptis laxiflora* flower extract: (a) low‐magnification image showing homogeneous distribution and quasi‐spherical morphology with particle sizes ranging from 45 to 110 nm; (b) high‐resolution image revealing lattice fringes with an interplanar spacing of approximately 0.113 nm, indicating the presence of localised graphitic domains.

High‐resolution transmission electron microscopy (HR‐TEM) analysis was performed to investigate the structural characteristics of the synthesised carbon dots. The low‐magnification image (Figure 3a, 500 nm scale) reveals that the particles are homogeneously distributed over a wide area and are clearly separated from each other. The particles predominantly exhibit spherical or quasi‐spherical morphology, which is consistent with the typical structural features expected for carbon dots. Particle size analysis performed using ImageJ indicated that the particle sizes range from 45 to 110 nm. Representative measurements of 45, 52, 69 and 110 nm further support this distribution. The high contrast and well‐defined particle boundaries confirm that the nanoparticles are well‐dispersed without noticeable aggregation, indicating the formation of a stable nanoparticle system.

To further elucidate the structural features, a single particle was examined at higher magnification (Figure 3b, 5 nm scale). The HR‐TEM image reveals distinct periodic atomic stacking patterns within the particle. Measurements performed using ImageJ showed that the average interplanar spacing of these ordered regions is approximately 0.113 nm. This result indicates that the carbon dots are not entirely amorphous; instead, they contain localised graphitic or crystalline‐like domains exhibiting short‐range order. Such structural ordering is a critical factor contributing to the characteristic optical properties of carbon dots. Overall, the HR‐TEM findings strongly support that the synthesised material possesses the necessary structural features for advanced optical and biomedical applications, highlighting its potential as a functional nanoplatform.

### 3.2 Fungal Growth Rate and Morphology Analysis

**FIGURE 4:**
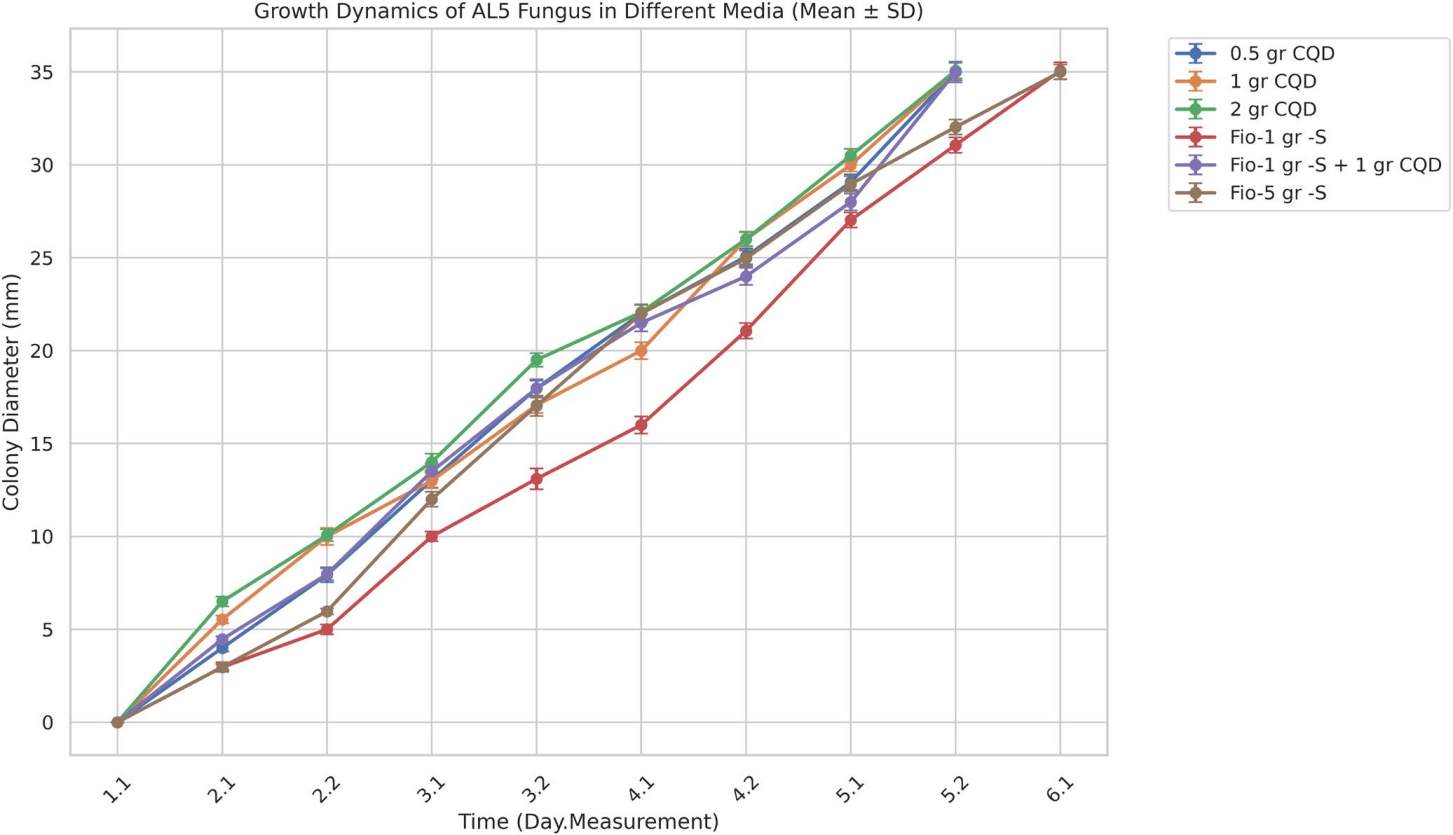
Growth dynamics of the AL5 fungus in different nutrient media with and without carbon quantum dots (CQDs). Colony diameter (mm) was monitored over a 6‐day incubation period and recorded at two time points per day when measurements were available (Day. Measurement). The tested conditions included Fio‐5 g ‐S, Fio‐1 g ‐S, Fio‐1 g ‐S supplemented with 1 g CQD and CQD‐enriched media containing 0.5, 1 and 2 g CQD. Data are presented as mean ± standard deviation (SD) from three independent replicates (*n* = 3), and error bars indicate the within‐group variability at each time point. Overall, CQD supplementation was associated with a faster increase in colony diameter during the early incubation phase compared with the −S controls, leading to an earlier approach to the maximum measurable colony size in CQD‐containing media, whereas the −S media displayed a comparatively slower growth trajectory.

In this study, daily growth rates of the AL5 fungus in different nutrient media were examined in detail. According to the data in Figure 4, the fungus growth rate and maximum growth level vary significantly depending on the nutrient medium used. The FIO‐5 g ‐S medium exhibited initially high growth rates compared to other media; however, by Day 6, the growth rate was restricted to 35 mm, indicating that this medium reached growth saturation after a certain period. Similarly, growth was observed from the beginning in the FIO‐1 g ‐S medium, reaching a maximum growth level of 35 mm in this medium as well.

On the other hand, nutrient media containing CQDs significantly enhanced the fungus's growth rate. Particularly in the FIO‐1 g ‐S and 1 g CQD medium, growth reached a maximum level of 35 mm by the second measurement on Day 5, demonstrating that CQDs had a stimulating effect on fungus growth and accelerated the development process. Additionally, rapid growth was observed in the medium containing 2 g CQD, reaching maximum growth levels by Day 5.

It was clearly observed that the addition of CQDs to nutrient media significantly increased the fungus's growth rate and allowed reaching maximum growth levels in a shorter period. The 0.5 g CQD and 1 g CQD media provided environments where the fungus exhibited controlled and continuous growth; however, they were not as effective as the 2 g CQD medium in terms of growth rate and achieved maximum values.

**FIGURE 5:**
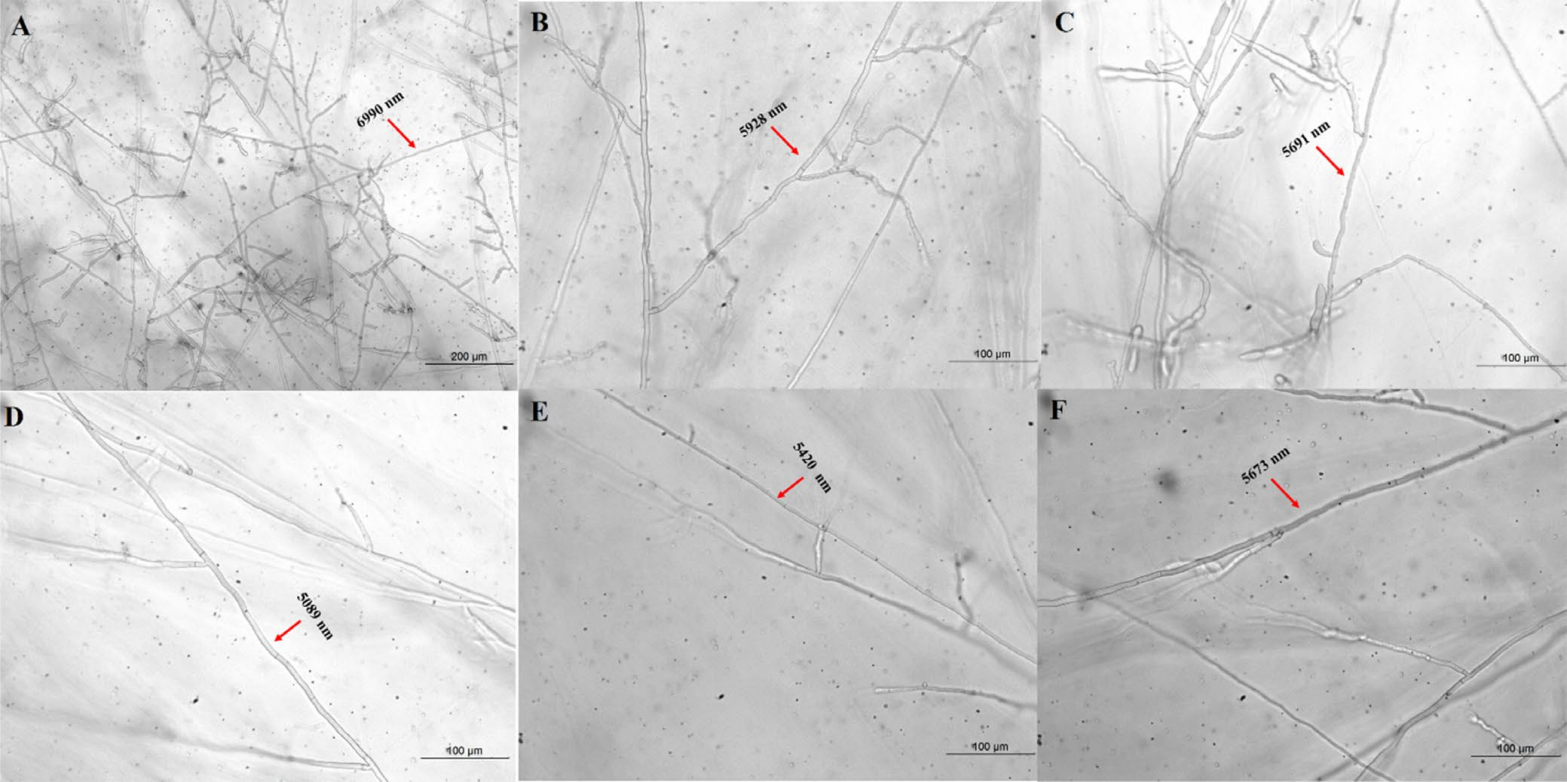
Hyphae diameters of different media (A) Fio‐5 g ‐S, (B) Fio‐1 g ‐S, (C) Fio‐1 g ‐S and 1 g CQD, (D) 0.5 g CQD, (E) 1 g CQD and (F) 2 g CQD.

The growth rates and hyphal diameters of the AL5 fungus in different nutrient media were examined (Table 2, Figure 5). According to the obtained data, the fungus exhibited the largest hyphal diameter (6990.10 ± 842.74 nm) and rapid growth in the FIO‐5 g ‐S medium. However, in this medium, the growth reached its maximum level by Day 6. This indicates that larger hyphal diameters allow cells to cover a wider area and uptake nutrients more effectively. In contrast, the growth rate of the fungus was relatively lower in the FIO‐1 g ‐S medium, and the hyphal diameter (5928.38 ± 542.72 nm) was smaller. This suggests that the cells expanded less and had more restricted access to nutrients.

**TABLE 2:** The growth rates and hyphal diameters of the AL5 fungus.

Medium	Fungus AL5 (nm)
Fio‐5 g ‐S	6990.10 ± 842.74^a^
Fio‐1 g ‐S	5928.38 ± 542.72^b^
Fio‐1 g ‐S and 1 g CQD	5691.30 ± 870.05^bc^
0.5 g CQD	5089.13 ± 802.84^d^
1 g CQD	5420.06 ± 738.93^cd^
2 g. CQD	5673.11 ± 638.98^bc^

*Note:* Values are expressed as mean ± SD. Different superscript letters (a–d) within the same column indicate significant differences among treatments (*p* < 0.05), whereas values sharing at least one common letter are not significantly different.

In the FIO‐1 g ‐S and 1 g CQD medium, the addition of CQDs decreased the hyphal diameter (5691.30 ± 870.05 nm) and increased the growth rate. In this medium, the fungus reached its maximum growth level by Day 5, indicating that CQDs contributed to more efficient growth by optimising cell expansion. In the 0.5 g CQD medium, the hyphal diameter (5089.13 ± 802.84 nm) was amongst the lowest, suggesting that the cells had a denser structure which supported rapid growth. Similarly, in the 1 g CQD medium, the addition of CQDs reduced the hyphal diameter (5420.06 ± 738.93 nm) and increased the growth rate of the fungus. The 2 g CQD medium supported rapid growth, and the hyphal diameter (5673.11 ± 638.98 nm) remained relatively small. This indicates that a high concentration of CQDs made the cell structure more compact, supporting rapid growth.

Overall, the effect of CQDs on fungal growth was clearly observed. CQDs enable faster fungal growth by reducing hyphal diameter, which is associated with a more compact cell structure and faster access to nutrients. These findings suggest that CQDs have the potential to be used as an important component in fungal biotechnology and industrial applications to enhance growth rates.

## 4 Discussion

Beneath the soil, arbuscular mycorrhizal fungi establish extensive hyphal networks that physically interconnect the roots of a variety of host plants. A single gramme of soil can contain tens to hundreds of metres of hyphae, which act as conduits for nutrient transport (Gadd and McGregor 2024). These networks, commonly referred to as common mycorrhizal networks, have significant effects on nutrient cycling and facilitate the global transfer of up to 5 billion tons of carbon annually. Associations with mycorrhizal fungi have played a crucial role in facilitating major evolutionary events worldwide, driving the evolution of intricate root structures and providing habitats for a broad spectrum of organisms (Guirguis et al. 2023).

Previous research has demonstrated that mycorrhizal fungi influence the success of their host plants by affecting which plants survive and reproduce (Baba and Hirose 2024). The fungi accomplish this by creating a complex hyphal network that explores the soil and accesses mineral nutrients such as phosphorus and nitrogen, which are exchanged for carbon compounds provided by the host plant. The transfer of resources from the fungus to the host plant is highly variable and is influenced by factors such as soil nutrient availability, host species, host age, light availability and even the host's sex (Guirguis et al. 2023). Because mycorrhizal networks can connect to multiple plants simultaneously, the transfer of resources through a common fungal network can vary depending on these factors (Long et al. 2022; Zhao et al. 2024).

Orchid mycorrhizal collaboration plays a crucial role, particularly in the germination and early development processes of orchids. Mycorrhizal fungi support this process by providing essential carbohydrates and other nutrients necessary for the successful germination and growth of orchid seeds. A study by Aytar and Kömpe (2023) on the effects of the AL5 mycorrhizal fungus on *Anacamptis laxiflora* presents significant findings in understanding orchid mycorrhizal interactions. In the study, *A. laxiflora* seeds were embedded in fresh hazelnut leaves and incubated for 28 days. During this period, under the influence of the AL5 mycorrhizal fungus, the seeds germinated and developed into young plant tissues known as protocorms. Protocorms are the foundational structures formed from the germination of orchid seeds, where root and leaf tissues first begin to develop. At the end of the 28‐day development period, the protocorms were observed to have reached approximately 1 mm in size. After the incubation period, by the 5th week, the protocorms progressed to a more advanced developmental stage, forming seedlings. At this stage, the seedlings had reached an average size of 1 cm, and root development had begun. By the 8th week, the seedlings had matured further and were ready to be transplanted into nature. In conclusion, the AL5 mycorrhizal fungus was found to play a critical role throughout the entire process from the germination of *A. laxiflora* seeds to the adaptation of the seedlings to their natural environment. This study underscores the positive impacts of mycorrhizal fungi on orchid species and highlights the importance of symbiotic relationships (Aytar and Kömpe 2023).

CDs offer significant potential across a wide range of applications such as energy efficiency, bioimaging, biosensors, lasers and light‐emitting diodes. These nanomaterials also hold promise as next‐generation fluorescent markers and can be utilised for high‐efficiency catalyst design in biomedicine and energy technologies. The properties of carbon dots make them ideal for various research and industrial applications (Pete et al. 2023).

For instance, Diao et al. (2018) synthesised blue and green, fluorescent CDs (B‐CDs and G‐CDs) using *Syringa oblata* Lindl and investigated fluorescent CDs with adjustable emission properties. B‐CDs were obtained via a hydrothermal process at 200°C for 4 h, whilst G‐CDs were synthesised by adding sodium hydroxide (NaOH) to the precursor solution. B‐CDs showed a size distribution ranging from 1.0 to 5.0 nm, whereas G‐CDs ranged from 2.0 to 8.0 nm. Additionally, B‐CDs exhibited a quantum yield of 12.4%, which was higher compared to 6.5% for G‐CDs (Diao et al. 2018).

In another study, Atchudan et al. (2018) investigated CDs derived from *Magnolia liliiflora* flowers. These CDs had an average size of 4 ± 1 nm and a quantum yield of 11% (Atchudan et al. 2018). Similarly, Wan et al. (2019) explored CDs synthesised from *Abelmoschus manihot* (Linn.) flowers, which demonstrated strong blue emission with a fluorescent quantum yield of 30.8% (Wan et al. 2019). These studies highlight the optical and chemical properties of CDs obtained from different plant sources and their potential applications.

The results obtained in the present study are primarily based on phenotypic parameters, namely colony diameter expansion and changes in hyphal diameter. Nevertheless, the growth‐promoting effect of AL‐CQDs on *Ceratobasidium* sp. may be interpreted within the framework of mechanisms reported in recent years regarding the interactions between carbon‐based nanomaterials and microorganisms. Carbon quantum dots possess abundant surface functional groups, including carboxyl, hydroxyl and amino moieties, as well as redox‐active defect sites. These structural features enable their participation in both surface‐mediated interactions and electron transfer processes in biological environments. Previous studies have demonstrated that carbon‐based nanomaterials, particularly in bacterial systems, can facilitate extracellular electron transfer (EET), thereby enhancing metabolic activity, supporting ATP production and improving substrate utilisation efficiency (Yang et al. 2020; Zhang et al. 2022).

Although the physiology of filamentous fungi differs from that of bacteria, hyphal extension and biomass accumulation are likewise strongly dependent on mitochondrial respiration and the maintenance of cellular redox balance. In this context, it may be proposed that AL‐CQDs indirectly modulated redox‐related processes in fungal cells, thereby enhancing metabolic efficiency and supporting accelerated growth. In addition, the biphasic dose–response model known as hormesis whereby nanoparticles may exert stimulatory effects at low concentrations and inhibitory effects at higher concentrations is well documented in the nanomaterial literature (Iavicoli et al. 2018; Gao et al. 2024). In the present study, the accelerated colony expansion observed particularly in media supplemented with 1 g and 2 g CQDs suggests that, below a potential toxicity threshold, CQDs may function as growth‐promoting agents. Although no evidence of growth inhibition was detected in our experimental conditions, the presence of a dose‐dependent response raises the possibility of a hormetic effect.

In conclusion, although the underlying mechanisms responsible for the phenotypic changes observed in this study were not directly elucidated, processes reported in the literature such as facilitation of electron transfer, modulation of cellular redox balance, surface‐mediated interactions and hormetic dose–response effects may be considered plausible mechanisms that could account for the growth‐accelerating impact of AL‐CQD application on fungal development.

## 5 Conclusions

This study demonstrated that CQDs promoted fungal growth, as reflected by accelerated colony expansion and a reduction in hyphal diameter. The decrease in hyphal diameter may suggest a more compact hyphal organisation and potentially more efficient nutrient utilisation, although the underlying mechanism remains to be clarified. Although CQDs are commonly investigated for their antifungal or antimycotic activities, the present findings indicate that, under specific conditions, they may also exert growth‐promoting effects.

In addition to their cost‐effective and environmentally friendly synthesis, CQDs may represent promising nanomaterials for modulating fungal development. However, the molecular basis of this stimulatory effect remains unresolved. Future studies should therefore focus on redox homeostasis, reactive oxygen species (ROS) dynamics and metabolic activity in order to elucidate the biological mechanisms underlying the observed enhancement in fungal growth.

## Author Contributions

**Idara Asuquo Okon:** writing – original draft, writing – review and editing, resources, investigation. **Yasemin Özdener Kömpe:** writing – original draft, writing – review and editing, validation, investigation, conceptualization, data curation. **Mika Sillanpää:** writing – original draft, writing – review and editing, investigation, software. **Erdi Can Aytar:** conceptualization, investigation, funding acquisition, writing – original draft, supervision, resources, validation, methodology. **Major Mabuza:** writing – original draft, writing – review and editing, resources, investigation. **Bengisu Sentürk:** conceptualization, investigation, writing – original draft, methodology, formal analysis, project administration, resources.

## Disclosure

During the preparation of this work, the author(s) used ChatGPT (OpenAI) to improve the clarity and language of the manuscript.

## Conflicts of Interest

The authors declare no conflicts of interest.

## Data Availability

The data that support the findings of this study are available on request from the corresponding author. The data are not publicly available due to privacy or ethical restrictions.
